# Psychological Stress Experienced by First-Year Medical Undergraduates: A Cross-Sectional Study From Eastern India

**DOI:** 10.7759/cureus.46725

**Published:** 2023-10-09

**Authors:** Sumana Panja, Arkadeep Dhali, Bhagyalakshmi Avinash, MunMun Chattopadhyay, Kankana Bhowmick, Jyotirmoy Biswas

**Affiliations:** 1 Physiology, Barasat Government Medical College & Hospital, Kolkata, IND; 2 Internal Medicine, Sheffield Teaching Hospitals NHS Foundation Trust, Sheffield, GBR; 3 Gastroenterology, University of Sheffield, Sheffield, GBR; 4 Orthodontics and Dentofacial Orthopaedics, JSS Dental College and Hospital, Mysore, IND; 5 Physiology, Tamralipto Government Medical College and Hospital, Tamluk, IND; 6 Psychology, Indian Institute of Science Education and Research, Mohali, IND; 7 Internal Medicine, College of Medicine and Sagor Dutta Hospital, Kolkata, IND

**Keywords:** psycho- social functioning, academia, medical students, mental stress, medical education

## Abstract

Background

While medical education is vital for producing competent physicians, its rigorous curriculum can harm students' mental well-being. This study focuses on assessing psychological stress in first-year medical students in Eastern India and aims to identify its primary causes.

Methods

This cross-sectional study involved 125 first-year MBBS students in a tertiary care medical teaching institution in eastern India. They completed the Medical Student Stressor Questionnaire (MSSQ-40) questionnaire to measure stress and provided academic records to be reviewed.

Results

Among the 125 students included in the study, male students demonstrated greater academic and interpersonal stress. The findings revealed that a substantial proportion (79%) of the student population experienced high to severe levels of academic stress, followed by 88% who reported moderate to high levels of social-related stress. Furthermore, it was observed that those students who experienced high to severe stress across all six domains tended to perform poorly during the initial half of their academic year.

Conclusion

The high levels of stress experienced by medical students can have significant implications for their academic performance. However, the nature of our study limits us to only highlight the existence of a correlation between the two. Future studies on the same should be conducted to assess the causal relation between these factors.

## Introduction

Medical education serves as a crucial foundation for the acquisition of knowledge, skills, and professionalism necessary for graduates to become proficient physicians. However, the demanding nature of medical training, characterized by curricular and environmental stress, can exert deleterious effects on the mental and emotional well-being of medical students, thereby posing a potential threat to the cultivation of these essential attributes [1]. The experience of stressors during the early stages of medical education, when students are adapting to the rigors of their training, further influences this dynamic.

Curricular stress arises from the extensive academic load, rigorous examinations, and the need to meet stringent performance standards inherent in medical education. The constant pressure to excel academically while absorbing vast amounts of complex medical information can contribute to heightened levels of stress among students. Environmental stress, on the other hand, encompasses factors such as a highly competitive learning environment, long working hours, limited personal time, and the emotional toll of dealing with patients and witnessing human suffering. These stressors, combined with the inherent challenges of adjusting to a demanding medical school environment, can adversely impact the mental and emotional well-being of medical students [2].

To counteract the negative consequences of stressors, it is paramount for first-year medical students to acquire effective stress management techniques. Developing the capacity to recognize and manage stressors can empower students to effectively cope with the demands of medical education while preserving their mental and emotional health. Techniques such as mindfulness exercises, time management strategies, regular physical activity, social support networks, and seeking professional assistance when necessary can contribute to a healthier and more resilient experience in medical school.

Moreover, beyond managing stress, the cultivation of qualities and values associated with leadership among medical students holds immense potential for creating a positive impact on society. Leadership traits such as effective communication, empathy, ethical decision-making, and collaboration are integral to the practice of medicine and the provision of patient-centered care. By embodying these qualities, medical students can not only enhance their own personal and professional development but also inspire and influence their peers, colleagues, and future patients [3]. 

## Materials and methods

This study employed an observational cross-sectional design to investigate stress levels among first-year medical students at the College of Medicine & Sagore Dutta Hospital, Kolkata, a tertiary care teaching hospital in eastern India. The study was conducted from October 2022 to November 2022 after obtaining approval from the institutional ethics committee (CMSDH/IEC/305/04-2022). The entire population of eligible students (125 first-year medical students) who provided consent were included in the study. At the time of data collection, the students were six months into the course. Students with pre-existing psychiatric disorders were excluded from participation.

Personal data pertaining to students' gender, fluency in the local language, medium of education prior to medical school, family income, number of family members, religion, and residential arrangement (hostel or with parents) were collected to assess social-related stress factors. A pre-validated MSSQ-40 (Medical Students Stress Questionnaire) questionnaire, originally developed in a medical school in Malaysia, was administered to measure stress and identify its sources among the students [3,4]. The questionnaire consisted of 40 items encompassing six domains of stressors elaborated in Table 1. 

**Table 1:** Domains of stressors.

	Type of stressor	Example of stressor
I	Academic-related stressors	extensive curriculum and time constraints, as well as tests and examinations.
II	Interpersonal-related stressors	conflict with peers, seniors, or teachers.
III	Teachings and learning-related stressors	inadequate learning resources and lack of proper guidance from teachers.
IV	Social-related stressors	peer pressure, adjusting to a new environment, hostel accommodations and food, and family responsibilities.
V	Drive and desires-related stressors	choosing the wrong professional course or fulfilling parental expectations.
VI	Group activities-related stressors	difficulty expressing oneself during group activities, introversion, and feelings of incompetence in group settings.

The MSSQ questionnaire was administered to the 125 consenting MBBS students in a classroom setting, with detailed explanations provided on how to complete it. Students with prior psychiatric illness and a history of illicit substance abuse were excluded from the current cohort of students. After the first six months of their medical study period, stress levels were checked with the MSSQ questionnaires. The students also appeared for their first internal assessment, and the resulting academic performance data was collected. Following data collection, the initial stage analysis was conducted, and the data were entered into Excel sheets. Students who were experiencing high to severe stress (in any domain) were provided counseling by faculty and referred to the institution's clinical psychologist.

All items within the six stressor domains were combined to measure the overall stress levels experienced by the medical students. Stress levels were categorized as follows: mild stress indicated no or insignificant stress, moderate stress denoted a reasonable level of stress, and severe and high stress indicated significant emotional disturbances with or without disruptions to daily activities, respectively (Table 2) [4]. These categorizations were used in the index study by Yusoff et al. [4].

**Table 2:** Grade and score for the stressor domains.

Grade	Corresponding Score
Mild- Does not cause any stress on you.	0.00 – 1.00
Moderate- Reasonably causes stress on you. However, you can manage it well.	1.01 – 2.00
High- Indicates that it causes a lot of stress on you.	2.01 – 3.00
Severe- It disturbs your emotions badly.	3.01– 4.00

To assess the correlation between gender and differences in stress prevalence among subgroups, Spearman's rho analysis was performed. A p-value < 0.05 was considered statistically significant. Statistical analyses were conducted using SPSS v.20 (IBM Corp, Armonk, NY).

## Results

**Figure 1:**
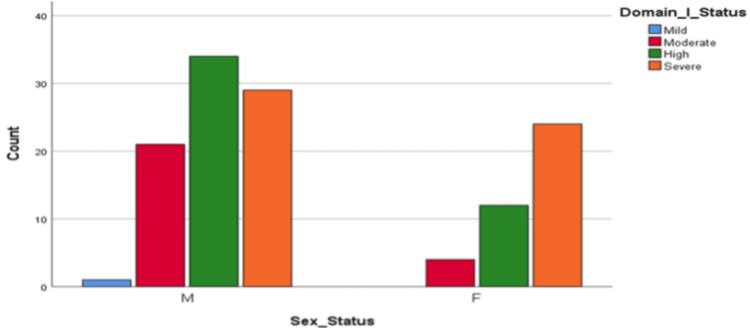
Gender difference in experience of stress in Domain 1 (p<0.05).

**Figure 2:**
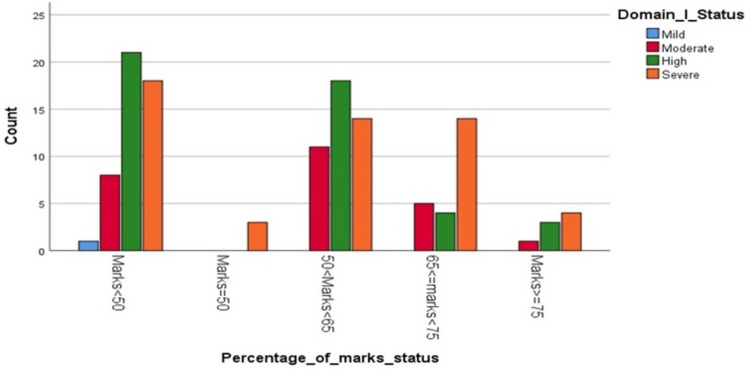
Difference in stress levels and in groups of different academic performance.

Based on a sample size of 125 students, it was observed that 85 (68%) were male and 40 (32%) were female. The domains that exhibited the highest levels of stressors were related to academic and social issues. Among these students, 98 (79%) experienced high to severe academic stress (Domain I), while 110 (88%) reported moderate to high levels of social-related stress (Domain IV). Domain-wise distribution of stress among the study population is shown in Table 3. It was found (Figure 1) that male students significantly suffered from academic and interpersonal stress compared to their female counterparts (p=0.042). Additionally, a correlation was observed between students experiencing high to severe stress in Domain 1 and their performance in the first half of the academic year, with a tendency to underperform (Figure 2).

**Table 3:** Domain-wise distribution of stress among the study population

Domains of stress	Percentage (95% CI) of moderate to severe stressed students in each domain	Median score (IQR)
Academic‐related stressors	93.8 (85.60‐96.02)	32 (23‐35)
Interpersonal and interpersonal‐related stressors	75.46 (65.21‐84.10)	10 (8‐15)
Teaching and learning‐related stressors	73.65 (65.10‐81.93)	09 (6‐11)
Social‐related stressors	73.40 (63.66‐80.93)	9 (6‐11)
Drive and desire‐related stressors	51.62 (40.85‐62.35)	3 (2-5)
Group activities‐related stressors	72.90 (61.09‐78.73)	7 (3‐8)

## Discussion

In the field of human psychology, a stressor refers to social and physical environmental factors that have the potential to influence an organism's abilities. When the body encounters a stressor, a complex cascade of hormonal and neurological processes is triggered, involving the hypothalamic-pituitary axis, which releases the hormone cortisol. Stress serves as a short-term adaptive mechanism to overcome immediate threats, but long-term or chronic stress can have detrimental effects on the body [5].

Research has indicated that even children attending primary school experience varying degrees of stress in the classroom. Given this understanding, it is reasonable to expect that the demands and challenges of the medical curriculum act as stressors for students [6]. While short-term stress can have positive effects on learning, it often negatively affects students' physiological and psychological well-being, particularly when stress becomes chronic [6]. Different students may perceive the same stressor differently based on their individual capacity to cope and combat stress [6].

Considering the significant impact of stress on learning, memory, and memory consolidation, it is crucial to comprehend the stressors students face and determine the most effective strategies to assist them in coping with these stressors [7]. The principal aim of this study was to evaluate the psychological stress levels among the targeted student population.

Based on the review of existing literature, there are identifiable stressors that are prevalent among medical students and contribute significantly to psychological stress. These stressors have been identified as the primary factors leading to psychological stress among medical students and they have been associated with potential consequences such as depression and burnout [8]. Consequently, students may experience adverse outcomes including impaired academic performance, cynicism, academic dishonesty, and maladaptive coping behaviors such as substance abuse. In extreme cases, these stressors may even contribute to suicidal ideation or attempts [9].

Therefore, it can be determined that academic challenges serve as the primary source of stress among medical students, which aligns with findings reported by various medical colleges across different geographical regions [9,10]. The considerable burden imposed by the curriculum and the limited time available contribute to high levels of stress, with 79% of students experiencing high to severe stress in Domain I. Notably, the most prominent stressors associated with academics encompass tests and examinations, time constraints, excessive study material, and falling behind in coursework [11-13].

A notable communication gap arises from the challenges faced by certain students in comprehending the language used in teaching [14]. Additionally, other social stressors encompass the adjustment to a new environment, disparities in cultural practices, and the integration into new peer groups. A high score in Domain IV indicates that students encounter difficulties in engaging in social and community activities. A study conducted in Malaysia also identifies similar issues, attributing the high-stress levels to challenges in adapting to the new social and educational environment, as well as the increased load of the curriculum [4].

Our research indicates that a significant proportion (52.2%) of students in medical schools experience mild to moderate stress. However, beyond the scope of our study, it may be due to uncertainties surrounding their expectations and demands compared to their high-school preparation. Addressing this issue, it is crucial to provide special attention to students' socialization and the role model status of their teachers [10].

Furthermore, a substantial percentage (63.3%) of students in our study reported experiencing moderate to severe stress, largely attributed to their high self-expectations. The limited number of available seats in postgraduate programs has intensified competition, adding to their stress levels.

The findings indicate that students are facing significant academic, social, and interpersonal stress [15]. As mentors and educators, it is incumbent upon the faculty members to work towards alleviating this stress and promoting their overall well-being [16]. A primary step in this process is recognizing the presence of stress and identifying its contributing factors.

To achieve this, a well-designed curriculum and schedule should be developed, aimed at maximizing knowledge acquisition while minimizing adverse stress. Creating a nurturing and supportive learning environment is equally important [17]. Faculty and students can foster good interpersonal relationships outside the classroom through institution-sponsored cultural and social events [18].

Taking a proactive approach, teachers can identify underperforming students and provide them with additional help and resources [19]. Establishing mental health clinics and programs within the institution has been proven to be highly beneficial for students' thriving and overall success [20].

In cases where mental health stigma may hinder seeking help, arranging out-of-institution mental health management can be beneficial [21]. It is also crucial to teach students healthy coping mechanisms to manage stress effectively. Peer discussion groups have shown promise in helping students analyze and address their stress and unique challenges [20].

Educators and teachers can play a significant role in supporting students by being aware of their needs and taking proactive measures to address stress and promote their well-being in the academic setting [21].

Limitations

Our study is not without its constraints. Chief among these is its cross-sectional survey design, conducted exclusively within a specific regional medical teaching institution in India. While very few investigations have explored related subjects, none have specifically delved into the nuances of this topic within the context of India's socio-economic milieu. To enhance the generalizability of our findings, it is imperative that future research encompass a broader spectrum of regions across India. Secondly, we have excluded students with pre-existing psychiatric illnesses from the study. Thirdly, the MSSQ-40 questionnaire was prevalidated in Malaysia. This has to be prevalidated in the Indian context for an accurate outcome. One can also argue that poor-performing students were stressed more rather than the other way around. Another finding from the MSSQ-40 questionnaire was that mild stress was calculated from zero. One can argue that while a corresponding score of 0.00 - 1.00 would technically qualify as minimal stress, a score of zero is likely invalid. It is highly improbable that a medical student would experience absolutely no stress at all, which means that anyone scoring a perfect zero is likely underreporting; the result of this would be inappropriate clumping of highly stressed, but underreporting individuals in our data. Hence consideration should be given for future studies to use a non-face-valid instrument to assess stress rather than a self-report questionnaire.

## Conclusions

The high levels of stress experienced by medical students can have significant implications for their academic performance. However, the nature of our study limits us to only point out that there is a correlation between the two. Future studies on the same should be done to assess the causal relation between them.
